# Disrupted functional brain connectivity networks in children with attention-deficit/hyperactivity disorder: evidence from resting-state functional near-infrared spectroscopy

**DOI:** 10.1117/1.NPh.7.1.015012

**Published:** 2020-03-11

**Authors:** Mengjing Wang, Zhishan Hu, Lu Liu, Haimei Li, Qiujin Qian, Haijing Niu

**Affiliations:** a Beijing Normal University, State Key Laboratory of Cognitive Neuroscience and Learning, Beijing, China; b Peking University Sixth Hospital, Institute of Mental Health, Beijing, China; c Peking University Sixth Hospital, National Clinical Research Center for Mental Disorders, Beijing, China; d Peking University, National Health Commission Key Laboratory of Mental Health, Beijing, China; e Beijing Normal University, Center of Social Welfare Studies, Beijing, China

**Keywords:** functional near-infrared spectroscopy, attention-deficit/hyperactivity disorder, functional connectivity, connectome, resting-state

## Abstract

**Significance:** Attention-deficit/hyperactivity disorder (ADHD) is the most common psychological disease in childhood. Currently, widely used neuroimaging techniques require complete body confinement and motionlessness and thus are extremely hard for brain scanning of ADHD children.

**Aim:** We present resting-state functional near-infrared spectroscopy (fNIRS) as an imaging technique to record spontaneous brain activity in children with ADHD.

**Approach:** The brain functional connectivity was calculated, and the graph theoretical analysis was further applied to investigate alterations in the global and regional properties of the brain network in the patients. In addition, the relationship between brain network features and core symptoms was examined.

**Results:** ADHD patients exhibited significant decreases in both functional connectivity and global network efficiency. Meanwhile, the nodal efficiency in children with ADHD was also found to be altered, e.g., increase in the visual and dorsal attention networks and decrease in somatomotor and default mode networks, compared to the healthy controls. More importantly, the disrupted functional connectivity and nodal efficiency significantly correlated with dimensional ADHD scores.

**Conclusions:** We clearly demonstrate the feasibility and potential of fNIRS-based connectome technique in ADHD or other neurological diseases in the future.

## 1 Introduction

Attention-deficit/hyperactivity disorder (ADHD) is a prevalent childhood-onset neurobehavioral disorder. Typical symptoms are age-inappropriate levels of inattention, hyperactivity, and impulsivity, which often lead them to dysfunctions in academic performance and social skills.^1^

Recent neuroimaging studies have demonstrated the disruption of functional or structural brain network organization in children with ADHD.^2^^–^^6^ According to the dual pathway model of ADHD, the main disruptions often occurred in the executive circuit or the reward circuit.^7^^–^^10^ Recently, evidences also suggest that visual network, which plays a key role in sustained attention,^2^^,^^11^ exhibits disconnection between the visual and other brain regions in children with ADHD.^12^^,^^13^ Furthermore, it has also been found that the brain network topology is altered in children with ADHD.^14^^–^^19^ For example, the ADHD patients exhibited decreased global efficiency and increased local efficiency compared to healthy individuals.^14^^,^^20^ These altered functional network characteristics were associated with various of clinical scores of ADHD or deficits in related cognitive functions.^9^^,^^12^^,^^13^^,^^21^^–^^23^ With these advances, however, the techniques are still frequently argued about complete body confinement and steadiness during brain scanning of children, especially involving children with ADHD due to their hyperactive characteristics.^24^

Functional near-infrared spectroscopy (fNIRS) is an optics-based brain imaging tool. It shows the advantages of high motion tolerance, few body constraints, and high portability.^25^ In recent years, fNIRS has been frequently used to explore the neural basis underlying different cognitive demands related to the ADHD, such as inhibition,^26^ working memory,^27^ cognitive flexibility,^28^ attention,^29^ and emotion regulation.^30^

Resting state is a natural imaging paradigm, and the resting-state fNIRS (rs-fNIRS) imaging has advantages over task-associated fNIRS.^31^ Due to its convenient operating procedure, rs-fNIRS can be easily operated in clinical practice, especially for child patients. Using rs-fNIRS, our group has demonstrated the feasibility,^32^ reliability,^33^^,^^34^ and reproducibility^35^ of this technique in characterizing functional connectivity and network topological properties. Furthermore, we and other groups have also demonstrated that rs-fNIRS technique can reveal the changes of brain network organization during normal development^36^^–^^40^ and under psychopathological conditions.^41^^–^^47^ These studies demonstrate that rs-fNIRS can be a promising tool in identifying disrupted brain networks in children with ADHD.

However, no rs-fNIRS study has been applied to explore the alterations in brain topological organization in ADHD children. To bridge this gap, we conducted an rs-fNIRS study with 30 ADHD patients and 30 healthy controls (HCs). As one of the neurodevelopment disorders, there is growing evidence from fMRI studies supporting both categorical and dimensional aspects of ADHD.^48^^,^^49^ Therefore, we hypothesized that the children with ADHD would exhibit aberrant network properties when compared to the HC group, which can assist the categorical diagnosis of ADHD. Furthermore, we hypothesized that these properties would be associated with dimensional ADHD scores.

## 2 Methods

### 2.1 Participants

Sixty participants were recruited for this study, which comprised 30 children with ADHD (boys, 7 to 12 years) and 30 sex-, age-, and education-matched HCs. The children with ADHD were recruited from Peking University Sixth Hospital, Beijing, China, and HCs were enrolled from a primary school in the local community. For the children with ADHD, the inclusion criteria were as follows: (1) a full-scale intelligence quotient (IQ)≥80; (2) right-handed; and (3) drug-naïve and free of other medical intervention. In addition, children with a diagnosis or history of head trauma with loss of consciousness, a history of neurological illness or other severe disease such as epilepsy, schizophrenia, pervasive developmental disorders (including autism spectrum disorders) or mental retardation were excluded.

The ADHD and comorbidities were diagnosed according to the DSM-IV criteria based on a semistructured interview using the clinical diagnostic interview scale^50^^,^^51^ by an experienced child psychiatrist. Accordingly, the children with ADHD can be categorized into inattentive type [ADHD-I, sample size (n)=22] and ADHD combined type (ADHD-C, n=8). Meanwhile, 25 of the children with ADHD exhibited comorbidities, such as disruptive behavior (n=8), anxiety disorder (n=2), mood disorder (n=3), tic disorder (n=6), and learning disorder (n=17). In addition, the DSM-IV symptoms of children with ADHD were scored using the ADHD rating scale-IV (ADHD RS-IV) to index the severity of ADHD.^52^ The items were rated on a four-point Likert-type scale (0 = never, 1 = sometimes, 2 = often, and 3 = always) by parents. Accordingly, the “inattentive,” “hyperactive/impulsive,” and “total” scores were computed for each child with ADHD.

This study was approved by the Ethics and Committee of Peking University Sixth Hospital/Institute of Mental Health. All subjects were treated according to the Declaration of Helsinki. Written informed consent was obtained from parents of the children. Meanwhile, the children aged above 10 years old also provided written informed consent by themselves. All the children provided oral consent before the experiment and were free to withdraw from the experiment at any time for any reason without prejudice to future care.

### 2.2 fNIRS Data Acquisition and Preprocessing

**Fig. 1:**
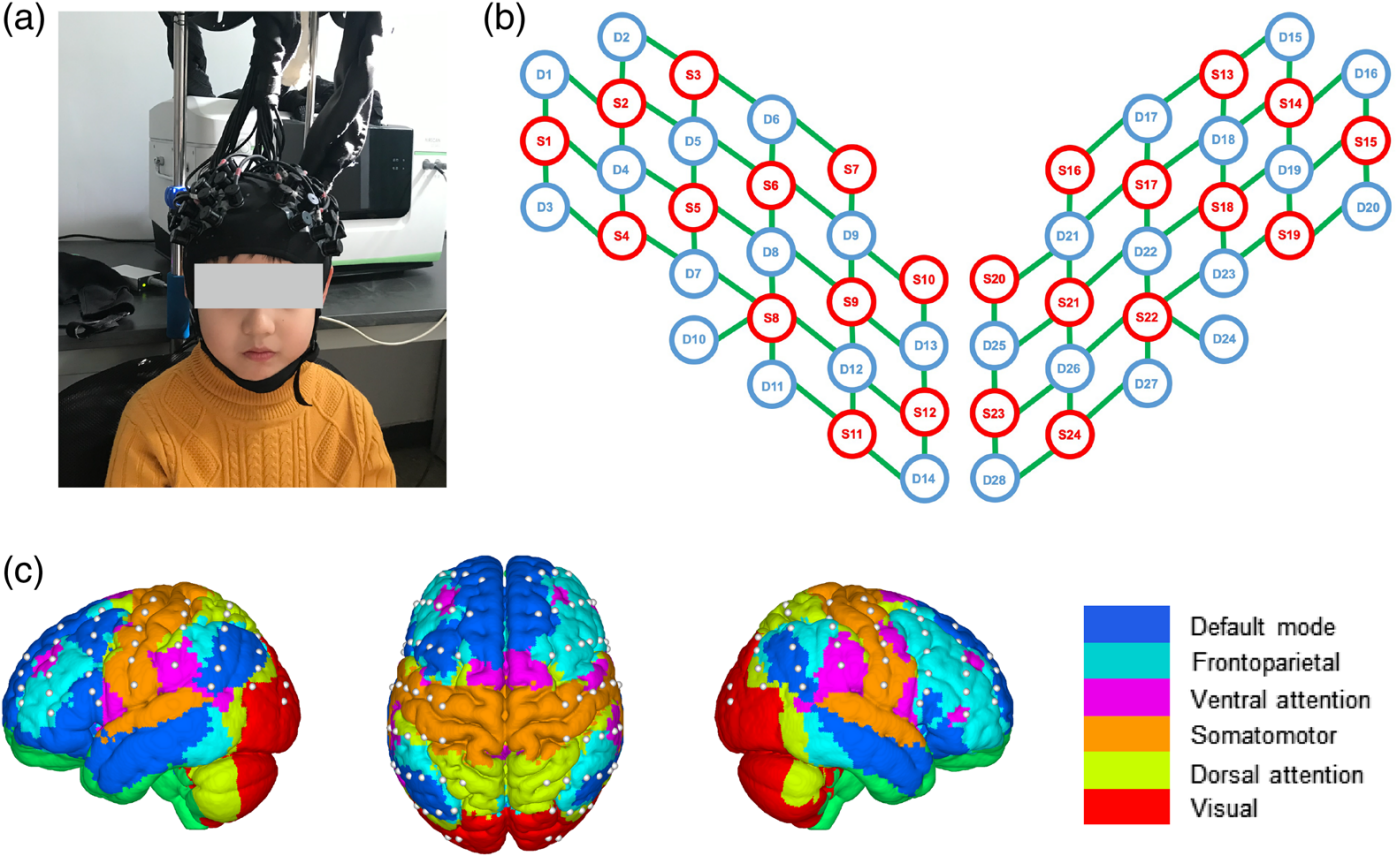
Schematic diagram of experimental data acquisition. (a) Photo obtained from a participant during the data collection. (b) Optodes and channels. The red circles represent the sources and the blue circles represent the detectors. Meanwhile, the green lines linking the sources and detectors represent the formed measurement channels. (c) The arrangement of the whole-head 80 measurement channels on a functional network brain template.^53^

fNIRS data were collected using a multichannel continuous wave near-infrared optical imaging system (Hui Chuang, China) with a sampling rate of 17 Hz. This system contains 24 light sources and 28 detectors. The optode arrays generated 80 different measurement channels with a fixed 3-cm interoptode distance, which covered primary regions of the whole head, e.g., frontal, temporal, parietal, and visual cortexes [Figs. 1(a) and 1(b)]. The optodes were placed according to the international 10–20 system, with the external auditory canals and vertex as the reference points. A resting-state fNIRS signal recording was lasted at least 12 min. During the recording, the participants were instructed to sit still and close their eyes without falling asleep. Such resting-state recording did not require overt perceptual input or behavioral output. Positions of the measurement channels were labeled by vitamin E capsules on an arbitrarily chosen participant, which were visible in the structural MRI imaging from a Siemens 3.0 Tesla scanner. According to the obtained spatial coordinates, these channels were displayed on Yeo et al.’s network template^53^ [Fig. 1(c)], in which six functional networks (i.e., the default, frontoparietal control, ventral attention, somatomotor, dorsal attention, and visual networks) were presented and labeled by different colors.

We used an in-house FC-NIRS package^25^ to preprocess the fNIRS data. Similar to our previous studies,^33^ a bandpass filter (0.01 to 0.1 Hz) was first conducted to eliminate the effects of low-frequency drift and high-frequency neurophysiological noise. Subsequently, we calculated the relative changes in the concentrations of oxygen-hemoglobin and deoxygen-hemoglobin using the modified Beer–Lambert law.^32^ Then we extracted 8 min stable hemoglobin time series for each participant. Finally, similar to our previous studies,^33^^,^^36^^,^^45^^,^^46^ we conducted a temporal independent component analysis to remove systematic physiological noise (e.g., superficial signal) and motion-induced artifacts.^54^ Specifically, these noise components were identified according to the components’ temporal profiles, spatial maps, and power spectra. A component would be considered noise if it met one of the following criteria: (1) the corresponding temporal profile included sudden jumps, slowly varied U or inverted U-shaped spike, or numerous intercurrent quick spikes (e.g., motion artifacts); (2) the dominant frequency of power spectra of the component was outside the range of 0.01 to 0.1 Hz; and (3) the spatial map of the component presented a global and spatially dispersive pattern (e.g., physiological interference). Once the noise components were identified, the concentration signal was subsequently reconstructed with these particular components eliminated from the original hemoglobin time course. The filtered concentration signal was used for further analysis. In this study, we used oxy-hemoglobin signal to present the following results because the HbO signal generally has a better signal-to-noise ratio than the HbR signal.^55^

### 2.3 Functional Connectivity Calculation and Brain Network Construction

For each participant, functional connectivity was calculated by conducting Pearson correlation analyses between time series of every pair of nodes, where the nodes were the measurement channels. This procedure generated an 80×80 correlation matrix for each participant. Of note, these correlation coefficients (r) were normalized to z-values with Fisher’s r-to-z transformation. With a predetermined sparsity that denotes the number of actual connections divided by the maximum possible number of connections in the network, the correlation matrix was then thresholded into a binary matrix that described the topological organization of the functional networks. As in our previous studies,^36^^,^^37^ we chose the sparsity of 0.2 to construct the brain network.

### 2.4 Brain Network Analysis

A graph theory method was used to characterize the topological organization of the brain functional networks in the ADHD and HC groups. Network measures were calculated using our FC-NIRS package.^25^ In fNIRS-derived brain network studies, topological network efficiency has been frequently used to characterize the capacity of parallel information processing within a brain network. We, therefore, focused on efficiency-related parameters, i.e., nodal efficiency, global efficiency, and local efficiency, to examine the differences in these efficiency measures between the ADHD and HC groups. The definitions for these parameters are described below.

The global efficiency $E_{\mathrm{glob}}$ represents the information transfer efficiency across the network, which is defined as the inverse of the harmonic mean of the shortest path length between any two nodes^56^

$$E_{\mathrm{glob}}=\frac{1}{N(N-1)}\underset{i\neq j\in G}{\sum }\frac{1}{d_{ij}},$$ (1)

where $d_{ij}$ is the shortest path length between node i and node j. Meanwhile, the local efficiency $E_{\mathrm{loc}}$ is defined as the average global efficiency of all subgraphs of the neighbors of node i ($G_{i}$)

$$E_{\mathrm{loc}}=\frac{1}{N}\underset{i\in G}{\sum }E_{\mathrm{glob}}(G_{i}).$$ (2)

In addition, for a given node i, its efficiency in information transfer is measured by $E_{\mathrm{nod}}$, which is defined as the harmonic mean of the shortest path length between this node and its neighbors

$$E_{\mathrm{nod}}=\frac{1}{N-1}\underset{i\neq j\in G}{\sum }\frac{1}{d_{ij}}.$$ (3)

### 2.5 Statistical Analysis

Two-sample t-tests were adopted to compare the differences in demographics or core symptoms between the ADHD and HC groups. For functional connectivity, a network-based statistic approach^46^^,^^57^ was adopted to compare the functional connectivity differences between the ADHD and HC groups. Specifically, two-sample t-tests with a threshold of p<0.001 were performed to identify the suprathreshold connections. These connections formed one or more subgraphs (components). Subsequently, 1000 permutations were performed to determine the significance of each component. Finally, the most significant component was selected to represent the altered functional connectivity. Furthermore, previous studies have found that the analogous regions in the resting-state network are strongly connected,^58^ and altered homotopic connectivity has been associated with many psychiatric conditions.^59^^–^^61^ In order to characterize the spatial attributes of the altered functional connectivity, we categorized the altered functional connectivity into three spatially different groups: (1) homotopic connectivity, denoting the interhemispheric connectivity between homologous regions; (2) intrahemispheric connectivity, denoting the connectivity between regions in the same hemisphere; and (3) heterotopic connectivity, denoting the interhemispheric connectivity that was not homotopic connectivity.^62^ For network efficiency, two-sample t-tests were also adopted to compare the differences between groups.

### 2.6 Relationship Between Altered Brain Functional Connectivity/Network Features and ADHD Core Symptoms

To test the associations between altered brain functional connectivity/network features and core symptoms (e.g., inattentive, hyperactive/impulsive, and total scores) in ADHD, Pearson correlation analyses were performed in the ADHD group with significance threshold of p<0.05. Before the correlation analyses, the effects of age, sex, and years of education were removed by multiple linear regression.

## 3 Results

### 3.1 Demographic and Core Symptoms

The t-test results for the demographic and core symptoms between the two groups are listed in Table 1. The ADHD and HC groups showed no significant differences in age or IQ. However, the children with ADHD exhibited significantly higher scores in core symptoms including inattentive, hyperactive/impulsive, and total scores (p<0.01) compared to the HCs.

**Table 1:** The demographic and clinical characteristics of children with ADHD and HCs.

	ADHD (n=30)	HC (n=30)	t value	p value
Age in month (mean±SD)	114.8±19.2	113.7±10.0	0.28	0.782
IQ (mean±SD)	109.8±12.9	115.7±12.6	−1.82	0.075
ADHD symptoms (mean±SD)				
Inattentive	16.1±2.9	8.6±4.7	7.52	<0.001
Hyperactive/impulsive	10.9±5.4	7.0±4.8	2.98	0.004
Total	27.0±6.6	15.6±8.7	5.73	<0.001

### 3.2 Decreased Functional Connectivity in Children with ADHD

**Fig. 2:**
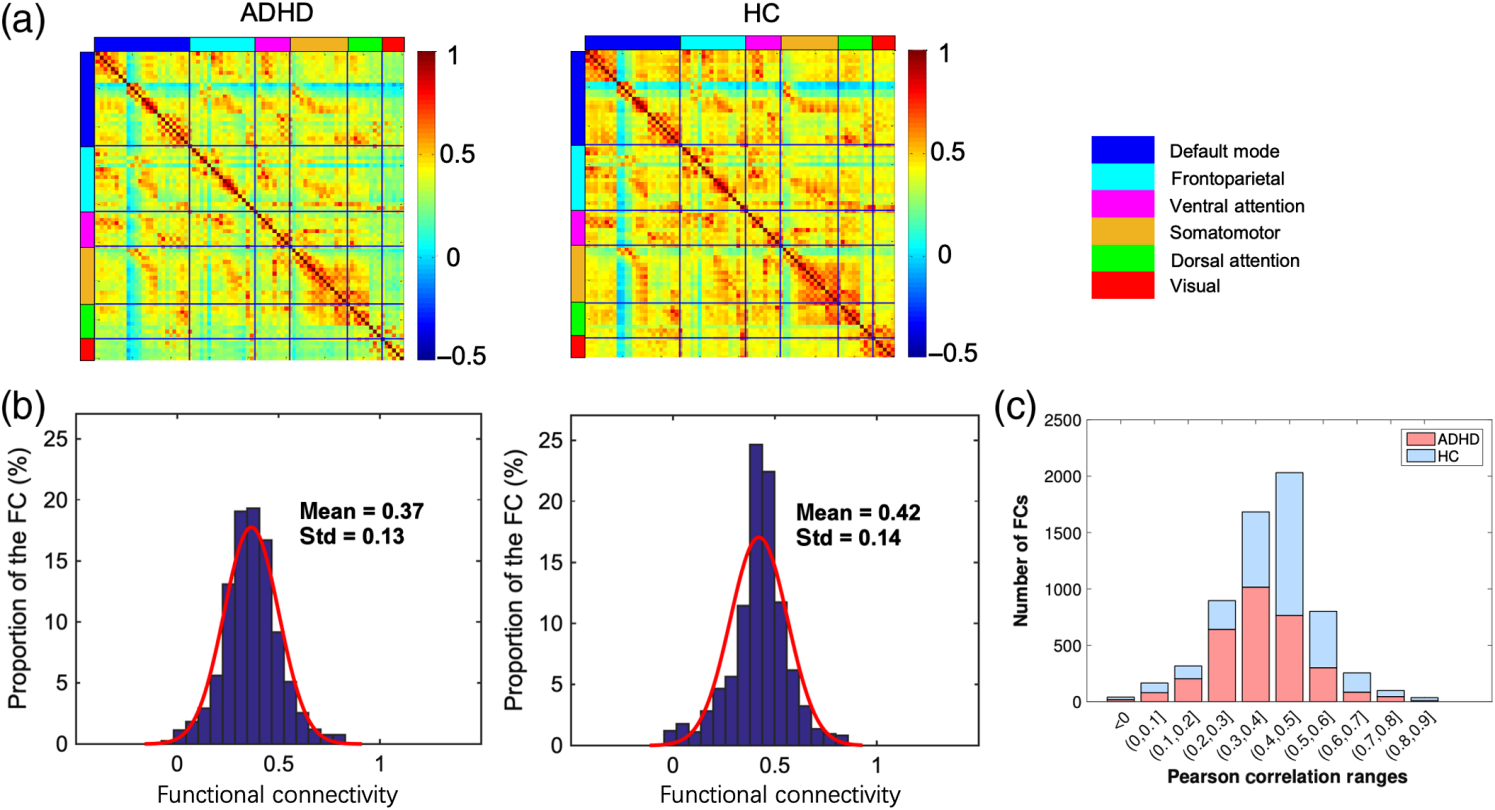
Spatial patterns of the functional connectivity in ADHD and HC groups. (a) Functional connectivity maps for these two groups. (b) Histograms of the functional connectivity distribution. The functional connectivity displayed approximately normal configuration in both ADHD and HC groups. (c) The stacked bar chart of functional connectivity across different thresholds.

Figure 2(a) shows the group-averaged connectivity strength in children with ADHD and HC groups. It was found that the averaged connectivity strength in ADHD was much lower (e.g., in somatomotor and dorsal attention networks) than that in HC although the spatial patterns of the functional connectivity maps between two groups exhibited obvious similarity. Quantitatively, the mean values of connectivity strength and its standard deviations were 0.37±0.13 for ADHD group and 0.42±0.14 for HC group [Fig. 2(b)]. Furthermore, the number of functional connectivity strength lower than 0.4 was much larger in ADHD group compared to that in HC group [Fig. 2(c)].

**Fig. 3:**
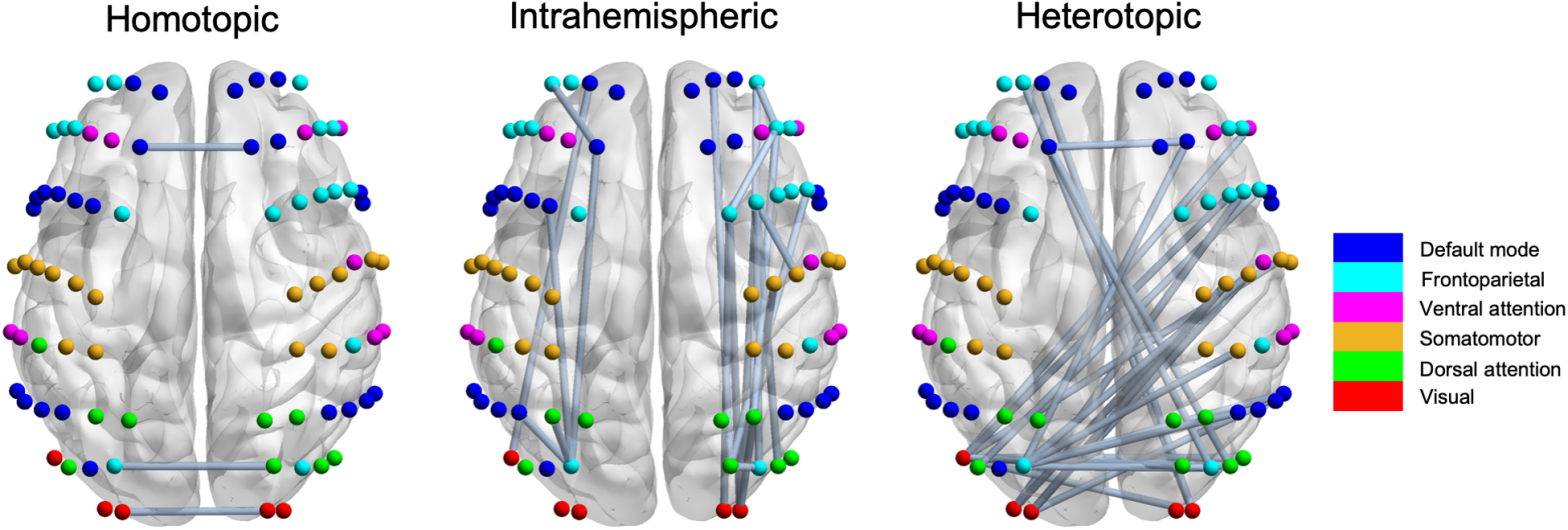
Significantly decreased functional connectivity in children with ADHD. The decreased functional connectivity was categorized into three groups: homotopic, intrahemispheric, and heterotopic connections. The dots represent measurement channels, and the colors label the cortical location of these channels.

Figure 3 shows the statistical differences in functional connectivity between ADHD and HC groups, in which significantly decreased functional connectivity was consistently found in ADHD group (p<0.05). Specifically, the changes in homotopic functional connectivity were mainly located in the default mode network, visual network, and between frontoparietal and dorsal attention networks. For intrahemispheric functional connectivity, the significantly altered connectivity was primarily centered in the right hemisphere involving the regions of default mode network, dorsal attention network and visual networks. For heterotopic functional connectivity, the changes were mainly located in the regions of frontoparietal and somatomotor networks in the right hemisphere and the regions of parietal and visual cortex in the left hemisphere.

### 3.3 Disrupted Brain Network Topology in Children with ADHD

**Fig. 4:**
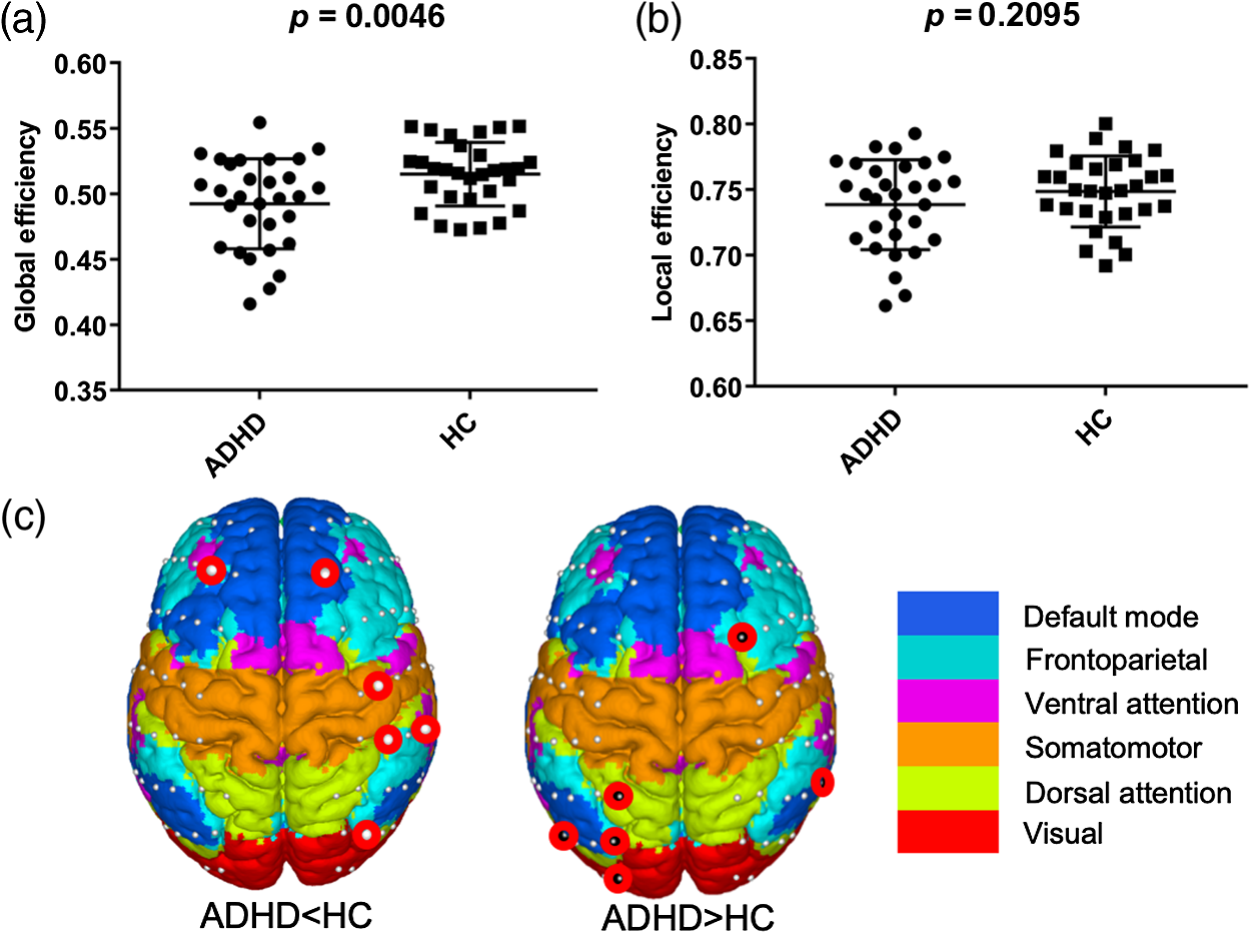
Group differences in (a) global, (b) local, and (c) nodal efficiencies. In (c), the red circles with white and black points indicate that the nodal efficiency significantly decreased and increased, respectively, in children with ADHD as compared to HC group.

For global network properties, the global efficiency in children with ADHD significantly decreased as compared to that in the HC group [Fig. 4(a)]. However, no significant difference was found in the local efficiency between two groups [Fig. 4(b)].

For regional nodal characteristic, the ADHD group exhibited both decreased and increased nodal efficiency in some primary brain regions [Fig. 4(c)] (p<0.05). Specifically, the decreased nodal efficiency was mainly located in the right hemisphere involving the somatomotor, default mode, and frontoparietal networks, and the increased nodal efficiency was mainly located in the left hemisphere involving the visual and dorsal attention networks.

### 3.4 Relationship Between Brain Network Features and Core Symptoms

**Fig. 5:**
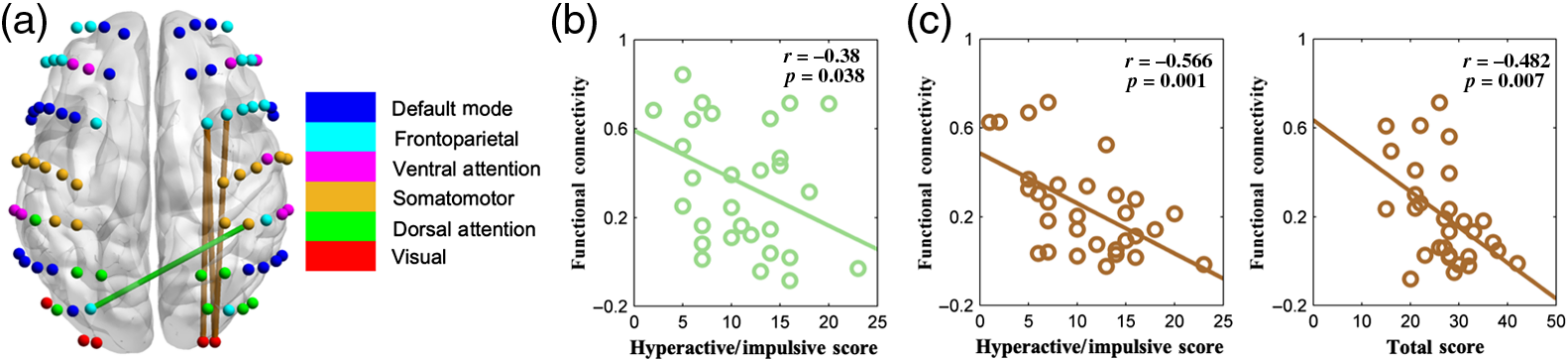
The relationship between functional connectivity and core symptoms in the ADHD group. (a) The connections showed significant correlation with core symptoms. (b) The scatter plotted between core symptoms and functional connectivity. (c) The scatter plotted between core symptoms and mean functional connectivity between right frontoparietal and visual networks.

Figure 5 shows the correlation relationships between functional connectivity and core symptoms. Four functional connections were found to be associated with the core symptoms. Specifically, functional connectivity that linked the left frontoparietal and right somatomotor networks [green line in Fig. 5(a)] showed a significantly negative correlation with the hyperactive/impulsive score in the children with ADHD [Fig. 5(b)]. The functional connectivity that linked the right frontoparietal and visual networks [brown lines in Fig. 5(a)] showed a significantly negative correlation with both the hyperactive/impulsive score and the total score in the children with ADHD [Fig. 5(c)].

**Fig. 6:**
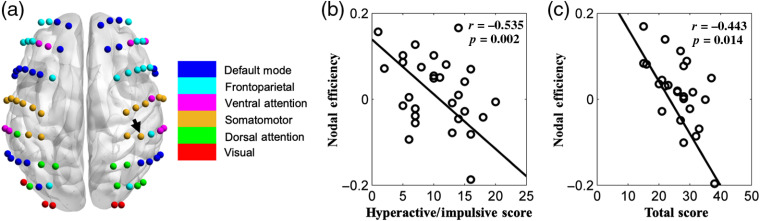
The relationship between nodal efficiency in the right somatomotor network and core symptoms.

For nodal efficiency, we identified one node in the right somatomotor network, as indicated by a black arrow in Fig. 6(a), showed a significantly negative correlation with both the hyperactive/impulsive and the total scores [Figs. 6(b) and 6(c)]. For the global and local network efficiency measures, no significant correlations were found between these features and core symptoms.

## 4 Discussion

The categorical–dimensional hybrid model of ADHD has provided insights to the pathophysiological mechanisms of this disorder in recent years.^48^^,^^49^ In this study, we used the rs-fNIRS and network analysis approach to study functional connectivity and topological network characteristics in the children with ADHD and the HC group, from the both categorical and dimensional perspectives. From the categorical perspective, we observed significant group differences as demonstrated by widespread reduction of functional connectivity strength, global network efficiency, and regional nodal efficiency in ADHD group. From the dimensional perspective, the disrupted functional connectivity and nodal efficiency significantly correlated with dimensional ADHD scores.

This study showed that children with ADHD, compared to the HCs, exhibited decreased homotopic, intrahemispheric, and heterotopic functional connectivity (i.e., disconnection). Specifically, the decreased homotopic connectivity was primarily located in the prefrontal cortices and bilateral posterior cortices involving dorsal attention networks and visual networks, which are, respectively, related to executive,^63^ attention,^64^ and visual sensory processing.^65^ Impairments in these cognitive processes have long been associated with ADHD.^66^ As such, our results are compatible with the previous findings and demonstrated the importance of the homotopic connectivity in cognitive functions;^59^^–^^61^ they further reveal that decreased homotopic functional connectivity impairs corresponding cognitive processes, which causes the ADHD symptoms. In addition, the disconnection between visual network and other cortical regions in our study is in line with the studies suggesting the potential important role of the visual network in ADHD.^12^^,^^13^ Furthermore, the disconnection between frontoparietal network and visual/attention networks in this study provides further evidence for the dual pathway model of ADHD,^7^ in which weaker regulation in the executive circuit from the frontal cortex to the visual and attention networks was identified. Moreover, previous studies also found that dysfunctions in the right prefrontal cortex were associated with ADHD.^26^^,^^67^

We also found decreased global efficiency in the ADHD group, which are consistent with the previous investigations from fMRI-derived network analysis in the patient group.^14^^,^^20^ In addition, we identified decreased nodal efficiency in the right hemisphere involving the default mode, somatomotor, and frontoparietal networks, as well as the increased nodal efficiency in the left hemisphere involving the dorsal attention and visual networks. According to the dual pathway model, it is reasonable to assume that the ADHD symptoms are associated with insufficient coordination between default mode, somatomotor, and frontoparietal networks and other brain regions, and the information overload in visual and attention networks.

The correlation results further confirmed the relationship between the functional connectivity network and ADHD symptoms. Specifically, decreased connectivity between the frontoparietal network and visual network was associated with increased hyperactive/impulsive and total scores in ADHD. This provides further evidence that the insufficient coordination between frontal cortex and visual networks may underlie the hyperactive/impulsive symptom in ADHD. In addition, the decreased nodal efficiencies in the right somatomotor network showed a negative correlation with the hyperactive/impulsive and total scores, which suggests that children with hyperactive/impulsive symptoms tended to have reduced information processing efficiency in the brain regions, i.e., somatomotor regions.

Despite the intriguing findings in our study, several issues need to be addressed. First, the approach adopted in this study should be applied to a larger sample to validate its robustness. Second, only the boys with ADHD were enrolled in this study, which limited the examination of the influence from gender differences on the current findings.^68^ Third, most of the participants in this study exhibited comorbidities. It is known that ADHD-related comorbidities would affect neurological change of the spontaneous brain activity of the patients.^69^^,^^70^ However, due to the relatively small sample size in this study, it remains unknown how the comorbidities would influence the current findings. Further sample collection in the future may provide better understanding of the potential confounding effects of comorbidities. Fourth, diagnosis of ADHD at an earlier age is critical for the earlier medical intervention, and applying our current approach to the infant and natal subjects benefits the diagnosis of ADHD. Last but not least, longitude investigation is highly preferable to reveal the neural development of ADHD.

It is noteworthy that the diagnosis of ADHD still heavily relies on the clinical information provided by parents and teachers and the ratings of the ADHD presentations.^71^ More quantitative biomarkers for ADHD benefit the diagnostic and therapeutic assessment of ADHD. Our current study validated rs-fNIRS as a potential tool in characterizing cortical network changes in patients with ADHD, which can serve as a potential biomarker for the diagnosis of ADHD.

## 5 Conclusions

In summary, we validated that the rs-fNIRS is a promising technique to characterize the topological network properties associated with ADHD. This study not only provides potential biomarkers for the diagnosis of ADHD but also has potential application for the investigation of neural basis underlying development, aging, and neurological diseases.
